# Emerging and Established Models of Bone Metastasis

**DOI:** 10.3390/cancers10060176

**Published:** 2018-06-01

**Authors:** Alexander H. Jinnah, Benjamin C. Zacks, Chukwuweike U. Gwam, Bethany A. Kerr

**Affiliations:** 1 Department of Orthopaedic Surgery, Wake Forest School of Medicine, Winston Salem, NC 27157, USA; ajinnah@wakehealth.edu (A.H.J.); cgwam@wakehealth.edu (C.U.G.); 2 Department of Cancer Biology, Wake Forest School of Medicine, Winston Salem, NC 27157, USA; bzacks@wakehealth.edu; 3 Wake Forest Baptist Comprehensive Cancer Center, Winston Salem, NC 27157, USA; 4 Virginia Tech-Wake Forest University School of Biomedical Engineering and Sciences, Winston Salem, NC 27157, USA

**Keywords:** bone metastasis, tissue engineering, mesenchymal stem cells, osteoclast, osteoblast, dormancy, mouse models, circulating tumor cell

## Abstract

Metastasis is the leading cause of cancer-related death and drives patient morbidity as well as healthcare costs. Bone is the primary site of metastasis for several cancers—breast and prostate cancers in particular. Efforts to treat bone metastases have been stymied by a lack of models to study the progression, cellular players, and signaling pathways driving bone metastasis. In this review, we examine newly described and classic models of bone metastasis. Through the use of current in vivo, microfluidic, and in silico computational bone metastasis models we may eventually understand how cells escape the primary tumor and how these circulating tumor cells then home to and colonize the bone marrow. Further, future models may uncover how cells enter and then escape dormancy to develop into overt metastases. Recreating the metastatic process will lead to the discovery of therapeutic targets for disrupting and treating bone metastasis.

## 1. Introduction

Bone is a common site of metastatic cancer, with an estimated 280,000 adults in the United States suffering from metastatic bone disease [1]. The cancers that most commonly metastasize to bone are prostate and breast cancer, which are also two of the most common cancers in the United States [2,3,4]. Additionally, lung, thyroid, kidney, and most adenocarcinoma primary tumors are reported to metastasize to bone, albeit less frequently [2,4]. These bone lesions cause serious skeletal complications, including spinal cord or nerve root compression, hypercalcemia of malignancy, pathologic fractures, and debilitating bone pain [1]. Furthermore, the median survival after a diagnosis of overt skeletal metastases is approximately 2–3 years [5,6]. These aforementioned facts illustrate the clinical importance of preventing or curing bone metastasis. Despite this, current treatment options for patients with bone metastases are seldom curative and are instead mostly palliative [2]. Further, metastatic bone disease poses a significant burden on the healthcare economy. Accordingly, Schulman et al. [7] estimated care for patients with bone metastases cost the United States $13 billion in 2005 alone. With the current emphasis on decreasing healthcare expenditure, a significant step towards a curative or preventive treatment for bone metastases would undoubtedly address a clinical and economic problem in one fell swoop.

The largest barrier to clinical translation in bone metastasis research is the lack of an appropriate in vivo animal model [8,9,10]. This lack is due to several factors, the most glaring being our incomplete understanding of the complex pathophysiological mechanisms at play during bone metastasis [2,9]. Increased knowledge of cancer cell osteotropism would be the foundation for the development of a more curative type of care. Therefore, the purpose of this review is to evaluate the current bone metastasis models and identify future directions for improvement.

## 2. Biology of Bone Metastasis

Stephen Paget first described a nonrandom pattern of metastasis to organs in 1889 while analyzing autopsy specimens of women who had died of breast cancer [11]. Paget developed the “seed and soil” hypothesis which compared disseminated cancer cells to seeds being dispersed while noting that plants will only grow if the seeds land in a congenial soil. In this example, osteotropic cells are the seeds, and the bone/bone marrow microenvironment acts as fertile soil for them to grow. Since the advent of the “seed and soil” hypothesis our understanding of metastatic mechanisms has significantly increased; however, this remains the backbone of the basic concept of cancer cell homing during bone metastasis.

Tumor metastasis is a multistep process consisting of tumor growth, angiogenesis, intravasation, survival in the circulation, and extravasation [6]. Tumors shed approximately 3.2 × 10^6^ cells/g tissue per day; however, only 0.01% of these cells survive the rigors of the systemic circulation and develop into metastases [12,13]. Furthermore, shed circulating tumor cells are predicted to comprise one cell out of 10^5^–10^7^ leukocytes in the bloodstream [14]. The cells that metastasize escape the primary tumor by releasing proteases. This allows them to cross the endothelium of small blood vessels, enter the circulation, and home to distant organs, including bone [2]. Bone is a common site of metastasis due to the high blood flow in the red marrow, presence of adhesive cells, mechanical support, and production of angiogenic and bone-resorbing factors that enhance tumor growth [2,10]. However, many of the factors that control the homing of circulating tumor cells to the bone remain to be discovered. One factor that has been shown to promote breast cancer cell bone colonization is receptor activator of nuclear factor-kappa B ligand (RANKL). In one study, osteoblast secretion of RANKL induced by the sympathetic nervous system enhances breast cancer cell homing and colonization [15]. Once cancer cells have survived the rigors of the systemic circulation, they invade the bone marrow and must possess certain phenotypic characteristics for overt bone metastasis to occur [2]. To colonize the bone, tumor cells must migrate across the sinusoidal wall which allows them to co-opt the hematopoietic stem cell (HSC) niche of the bone marrow. In doing so, these cancer cells compete with HSCs in the surrounding tissue, causing HSCs to evacuate the bone marrow. In addition, the cancer cells acquire the HSC’s mechanisms of proliferation and chemotaxis, which they previously used for blood cell production [16]. One way tumor cells home to and colonize bone is via the CXCL12/CXCR4 signaling axis. Receptor CXCR4 on cancer cells at the primary tumor site responds to CXCL12/Stromal-derived factor-1α, which is secreted into circulation by osteoblasts, inducing chemotaxis and further homing to and accumulation in the bone. The disseminated tumor cells must then survive, stimulate angiogenesis, and migrate to the bone surface. The tumor cells release signaling proteins, such as vascular endothelial growth factor (VEGF), parathyroid hormone-related peptide (PTH-rp), bone morphogenic protein (BMP), and wingless (WNT), that stimulate the displacement of osteoblasts lining the bone surface, activating bone resorption by osteoclasts, and allowing tumor cell infiltration of the surface of the demineralized bone [17]. However, the microprocesses that regulate the cancer cell movement and survival upon arrival at the distant organ remain elusive [6]. One mechanism that is theorized to contribute to the cell survival within bone is through the osteogenic niche [18]. Niche interactions are mediated by heterotypic adherens junctions, which activate the mTOR pathway leading to progression from single cells to micrometastases [18,19]. In both advanced breast and prostate cancer, there is about a 70% chance of the primary cancers metastasizing to bone [6]. However, for prostate cancer, most patients will die from other causes before overt bone metastases occur. This is due to the tendency of disseminated tumor cells to initially become dormant after colonizing the bone [6].

### 2.1. Dormant Lesions

One of the most perplexing mysteries surrounding metastatic disease is the concept of dormancy [20]. Dormancy is a phenomenon where disseminated tumor cells persist in a long-term state of quiescence and are eventually reactivated to induce metastatic relapse [21]. Reactivation can occur months to years after resolution of the primary tumor, with tumor cells remaining dormant within the bone marrow [22]. The presence of disseminated tumor cells in a patient with no evidence of disease puts the patient at a higher risk for relapse [22]. Metastatic dormancy has remained understudied in part due to the lack of appropriate animal models [21]. However, several genes have been identified to be implicated in the dormancy process, which includes BMP-7, transforming growth factor-beta2 (TGF-β2), BMP-3B, MSK1, and leukemia inhibitory factor receptor [23,24,25,26]. Furthermore, stable microvasculature promotes a dormant niche in breast cancer cells through expression of endothelial-derived thrombospondin-1 [20]. Once cancer cells are reactivated, lesions can either be osteolytic (bone destructive), osteoblastic (bone forming), or mixed. Breast cancer commonly results in an osteolytic metastasis (73%) while prostate cancer results in an osteoblastic metastasis (68%) [2,6,10]. Other advanced cancers (lung, melanoma, thyroid, kidney, and gastrointestinal) have demonstrated bone metastasis, but not with the same frequency.

### 2.2. Osteolytic Lesions

**Figure 1:**
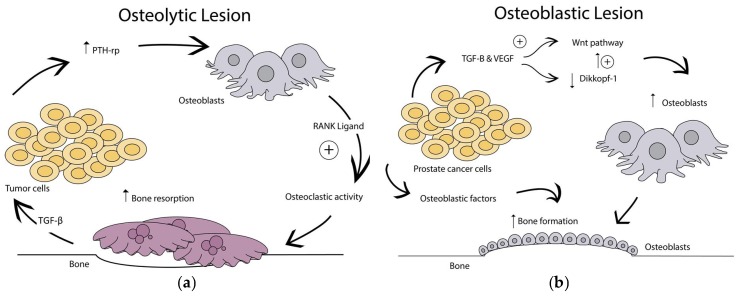
Bone metastatic lesions can be either osteolytic or osteoblastic. (**a**) Osteolytic lesions are caused by overactivation of osteoclast bone resorption; (**b**) Osteoblastic lesions result from direct tumor stimulation of osteoblasts. PTH-rp: parathyroid hormone-related peptide; RANK: receptor activator of nuclear factor-kappa B; TGF-β: Transforming growth factor-β.

Osteolytic lesions are caused by overactivation of bone resorption and can be identified on radiographs as lesions with decreased bone mineral density [27]. Disseminated tumor cells initiating metastatic lesions enter the bone surface by stimulating osteolysis via enhanced osteoclast differentiation [2]. Osteoclasts originate from hematopoietic precursor cells in the bone marrow and have a primary role of bone resorption [28]. Continued stimulation and loss of bone resorption regulation by osteoclast activation form the basis of an osteolytic lesion (Figure 1a) [29]. Anti-resorptive therapies (bisphosphonates) effectively reduce this cycle, thereby reducing pain and skeletal complications [30,31,32,33]. The most established growth factor in bone that contributes to osteolytic lesions is TGF-β [34]. It is theorized that TGF-β released by osteoclasts induces pro-osteolytic gene expression leading to PTH-rp proliferation from the cancer cells [35,36]. This increases osteoblastic production of RANKL, thereby indirectly stimulating osteoclast formation (Figure 1a) [37]. Cancer cells themselves can also produce RANKL, increasing osteoclast activation [38]. Continued bone resorption causes the release of more bone matrix proteins and growth factors that stimulate further tumor cell proliferation, leading to a cruel cycle of osteolysis [34]. Furthermore, TGF-β increases cyclooxygenase-2 expression, which correlates with an increase in interleukin (IL)-8. IL-8 induces osteoclast formation and activity independent of the RANK ligand pathway [39]. Additionally, monocyte chemotactic protein (MCP)-1 may play a key role in osteoclast differentiation and fusion in metastatic prostate cancer [27,30,40]. This continued breakdown of the bone structure contributes to the bone pain and pathological fractures experienced by patients with osteolytic bone metastases.

### 2.3. Osteoblastic Lesions

Osteoblastic lesions are characterized by increased bone formation. These can be identified on radiographs as increased areas of sclerosis within the skeleton [41]. Metastatic lesions from prostate carcinomas are the most well-known producer of osteoblastic lesions [2,29,42]. Osteoblasts originate from mesenchymal progenitor cells and function by forming bone. They do so by the stages of proliferation, matrix maturation, and mineralization [43]. The growth of prostate cancer cells alters bone remodeling by secreting factors that directly affect the osteoblast and osteoclast relationship (Figure 1b) [29]. The cancer cells produce RANK ligand and osteoprotegrin (OPG), thereby disrupting the balance in normal osteoclast activity [44]. Furthermore, there is an abundant release of TGF-β and vascular endothelial growth factor (VEGF) by the cancer cells, which directly affect the osteoblast activity [45]. This is done through the WNT pathway, which is implicated in osteoblastogenesis [46,47,48]. The combination of this WNT pathway upregulation coupled with the reported decreased expression of the WNT antagonist, dikkopf-1, in patients with advanced prostate cancer is associated with the formation of osteoblastic lesions (Figure 1b) [49]. Finally, the prostate cancer cells express large amounts of factors that strengthen the osteomimicry [50]. It is believed that the prostate cancer cells have this effect because the distant tumors induce osteoblast activation and bone formation prior to metastasis occurring as part of the preparation of the premetastatic niche [51]. Interestingly, pathology reports indicate that these osteoblastic lesions often form on an area of prior osteolysis in the premetastatic niche [52]. While areas of increased bone may seem beneficial, the inconsistent structure that results leads to unequal distribution of mechanical loads through the bone, producing bone fractures. In many patients, mixed lesions of osteolytic and osteoblastic sites increase the risk of fractures, and the structure of the bone becomes even more patchworked. How each type of lesion is initiated and progresses remains a mystery which will eventually be solved through new bone metastasis models.

## 3. In Vivo Models of Bone Metastasis

Our lack of understanding regarding bone metastasis stems directly from the fact that there are currently no suitable animal models to mimic human tumor cell metastasis to the bone microenvironment. The importance of in vivo studies in developing new therapeutic methods to combat the effects of metastatic disease cannot be understated. Prior to embarking on clinical trials in human patients, a new therapy must first be thoroughly tested in animal models [53]. However, the animal model used should reflect the environment that will be encountered in the human body. There are currently several in vivo models that exist to evaluate bone metastases; however, they all have their limitations [10,54].

### 3.1. Spontaneous Bone Metastasis

Spontaneous bone metastasis in animal models is currently nonexistent because this phenomenon is rare and difficult to recreate in most animal species [54,55,56]. However, a select few reports of metastatic disease in large animals (canine and feline) to bone have been reported [56]. There is a single report of lung adenocarcinoma in a feline species that underwent spontaneous metastasis to bone [57]. However, this is rare and does not present a feasible avenue for future research modeling. Canines are the only animal where prostatic cancers metastasize to bone reliably due to canine prostatic tissue undergoing similar changes to human tissues [56]. Despite this, the rarity and difficult identification do not allow suitable models to be recreated reliably [55,56]. Further, due to the small numbers of animals available and the cost of rendering care, large animal models are particularly unsuitable for initial testing of treatments. Thus, additional models were developed in rodents, but these models do not mimic the process of spontaneous metastasis. In the few rodents and larger animals in which spontaneous tumor initiation and metastasis does occur, the progression is slow, requiring months or years of tracking the animals; this timeline is prohibitive for testing therapeutic interventions. Thus, the field has focused on developing models of bone metastasis that will progress quickly and occur reliably in most animals. timeline is prohibitive for testing therapeutic interventions. Thus, the field has focused on developing models of bone metastasis that will progress quickly and occur reliably in most animals.

### 3.2. Intraosseous and Intracardiac Models

Another method of investigating the biological progression of tumor cells in a bone microenvironment involves direct implantation of cancer cells into the bone. This is done via injection of cells into the tibia or femur of a mouse, is termed an intraosseous model, and allows incorporation of the cells that can replicate tumor-induced changes in murine bone [58,59,60,61,62]. A series of intraosseous models are listed in Table 1. Direct injection into the bone microenvironment results in overt metastasis arising quickly, allowing testing of treatments for slowing or preventing metastatic growth. The limitation of this model is that it only resembles the final stages of bone colonization, preventing the study of homing, extravasation, and dormancy, and thus is more analogous to a primary tumor model [10].

To solve this problem and create a more metastatic model, some groups attempted intracardiac injection of osteotropic cancer cells to quickly induce bone metastasis at a high frequency [63,64,65,66,67]. Some current intracardiac injection models are listed in Table 2. In addition, tail vein injections may be performed to mimic hematogenous metastasis. Interestingly, there is only one model used that uses immunocompromised animals to investigate prostate cancer cell lines [67]. Other intracardiac models use Dunning rats and are discussed in Section 3.3 [68,69]. The xenograft models recapitulate extravasation and colonization, and the cells may undergo dormancy during the metastatic progression. Many of these models rely on human cell lines to study osteotropism. The use of a xenograft presented a major limitation in that, to avoid graft rejection, immune-compromised hosts were necessary. This eliminates the ability to examine the role of the immune system in tumor progression.

### 3.3. Immunocompetent Models

Due to the known link between the immune system and the skeletal system in cellular mechanisms, the science of “osteoimmunology” began to gain attention [70,71]. Osteoimmunology references the link discovered between T cell activation and bone resorption, particularly that seen with metastatic bone lesions [72]. The skeletal and immune systems share regulatory molecules; thus, disseminated tumor cells that act on the skeleton are affected by the immune system [72,73]. Tumor-specific cytotoxic CD8+ T cells participate in the killing of antigen-positive tumor cells, suggesting a protective role in metastatic dissemination [73,74,75]. Therefore, bone metastasis models were developed using immunocompetent mice for murine breast cancer, melanoma, and prostate cancer cell lines to allow for investigation of the effects the immune system may have on any potential treatments (Table 3) [76,77,78]. Furthermore, using the Dunning prostate cancer cell lines, a series of models using immunocompetent rats were developed [69,79]. These models represent a tremendous advancement in preclinical models of bone metastasis; however, most still require an intracardiac or intra-arterial injection of cancer cells. Although this is a reproducible technique, it would lead to obvious systemic issues that may affect the mechanisms being investigated within the bone [80]. Furthermore, this has limited translational applicability due to differences in species [9]. Most immunocompetent models require the injection of cells directly into the circulation and are not models of spontaneous metastasis. The models are useful in examining homing and colonization but cannot be used to study intravasation and premetastatic niche formation due to the lack of a primary tumor.

Due to the limitations discussed above, a novel model that recapitulates the metastatic process using 4T1 breast cancer cells was developed [81,82]. Cells were injected into the mammary fat pad and demonstrated spontaneous metastasis to lung and bones [81]. However, to our knowledge, this is the only model to be described using the orthotopic implantation method while using immunocompetent animals [80]. This is an exciting avenue of research for breast cancer, and the findings may apply to other tumors causing osteolytic metastases but will be less relevant to cancers with osteoblastic metastases, such as prostate cancer.

### 3.4. Humanized and Tissue-Engineered Models

Another alternative model growing in popularity is the use of a “humanized” model for metastasis [9]. These models aim to use human cancer cells and human bone implants to serve as the target for metastasis [83,84,85,86,87,88]. A list of humanized models can be found in Table 4. The metastatic progression method being examined in each study varies based on the injection technique. Humanized models attempt to recapitulate tumor progression in mice using human cells to better represent the process in patients. All models used a subcutaneous implant of human bone or a tissue-engineered construct. These models often still use direct injection of tumor cells into the circulation, but newer models may involve spontaneous metastasis from a primary tumor (orthotopic) [86,88,89]. For those using an intravenous or intracardiac injection technique, the authors are primarily investigating the ability of the cells to extravasate. Direct injection into the bone examines the cells’ ability to colonize within the bone microenvironment. Finally, with orthotopic models, the authors are investigating intravasation, survival in the circulation, and extravasation. However, the availability of human tissues is limited; therefore, several authors have implemented tissue engineering to create a reproducible and controllable microenvironment [10,89,90].

Tissue-engineered bone metastasis models, listed in Table 5, take advantage of recent advances in regenerative medicine to create a new bone microenvironment using scaffolds. The various scaffold materials provide structural support and environmental cues promoting osteoblast differentiation and function. Depending on the cells used to seed scaffolds, the entire heterogeneity of the bone marrow may or may not be represented. Nevertheless, current models incorporating this technique still rely upon an intracardiac injection and immunocompromised animals and, therefore, will be subject to systemic issues and a lack of immune response, as discussed previously [9].

### 3.5. In Vivo Dormancy Models

One final limitation to the current in vivo bone metastasis models revolves around the inability to recapitulate dormancy and homing [21,22]. Xenograft models have provided the minimal knowledge garnered on homing and dormancy. The basis of these models is that cell cycle arrest of cancer cells can be controlled and is reversible by either a change in microenvironment or by inhibiting signaling pathways [91,92,93]. There appears to be one attempt in the literature to incorporate dormancy into an in vivo model; however, this has only reliably recreated dormancy in some of the breast cancer lines investigated [91]. The authors used 3D biomatrices containing bone marrow stem cells and breast cancer cells (MDA-MB-231) and subcutaneously implanted these into NOD/SCID mice. After 24 h, either a supportive (DMSO) or inhibitory niche (activating receptor-like kinase inhibitors—SB431542, SB203580, and S1042) seeded 3D biomatrix was implanted on the contralateral side, and tumors grew within a supportive niche, but no tumors were found in the inhibitory niche. The authors demonstrated that cancer cells at the original seeding density were present within the inhibitory site, thus proving that the cancer cells did not proliferate nor die; therefore, the authors concluded that the remaining cancer cells were dormant. However, due to the paucity of research in this area, there is vast room for growth in the future.

## 4. Future Directions

Despite the push towards a focus on in vivo models by some, others believe that the ideal way to investigate the complex molecular mechanisms involved in this process is by advanced in vitro modeling [94,95,96,97,98,99,100]. These models consist of microfluidic models or advanced mathematical modeling, among others. 

### 4.1. Microfluidic Models of Metastasis

The general principle behind a microfluidic model is to recreate the 3-dimensional (3D) microenvironment of in vivo tissues, while also allowing the researcher to have complete control of the microenvironment [95]. This allows for metastatic migration from a 3D origin tissue to a 3D target tissue, within a controllable fluidic environment [95]. Four models for bone metastasis in a microfluidic model have been identified in the literature [97,101,102]. Bersini and Jeong [101,102] used a tri-culture system, consisting of osteo-differentiated human bone marrow (h-BM) mesenchymal stem cells (MSCs), endothelial cell monolayer, and human breast cancer cells (MDA-MB-231). With this model, the authors demonstrated that breast cancer cells extravasated into the bone microenvironment significantly more than a collagen control and that this increase in extravasation was associated with cross-talk between the h-BM MSCs and the MDA-MB-231 cells through CXCL5-CXCR2 paracrine signaling pathways [101]. The authors then refined this system by introducing human umbilical vein endothelial cells into the initial culture of the bone microenvironment to induce a microvascularized bone environment [102]. This allowed the authors to identify that the breast cancer cells responded to bone stromal cells through the aforementioned paracrine signaling, again leading to extravasation. Through the use of this novel model, the authors also identified that the myoblast cell line C2C12 had a protective effect against metastasis. Finally, the most recent microfluidic model to be introduced is from Hau et al. [97] The authors attempted to identify weak areas in the model presented by Jeong and Bersini and the main limitation to improve upon was to allow maturation and growth of the osteoblastic cell lineage, allowing mineralization and natural collagen fiber organization that may be involved in the complex underlying metastatic mechanisms. This was performed by using a miniaturized bone-on-a-chip model consisting of two compartments. The first of these allows for medium changes, while the second allows for osteoblastic tissue growth. The authors used MC3T3-E1 bone cells in a miniaturized bone-on-chip model with resultant spontaneous formation of thick, mineralized osteoblastic tissue. Furthermore, their co-culture with MDA-MB-231 and osteoblastic tissues demonstrated hallmarks of breast cancer colonization. While these microfluidic models lack some of the complexity of the in vivo models, including a functional immune system, they are ideal for high-throughput screening of potential therapeutics aimed at preventing or slowing metastasis. Despite the novelty and advances that can be made with these models, they are subject to limitations. Firstly, these models do not include immune cells. This is an important determinant, because the effects of the immune system on bone metastasis may be significant. Furthermore, none of these models include osteoclastic cell lines which we have identified as an important part of the metastatic process.

### 4.2. In Silico Models of Metastasis

Another method to identify potential therapeutic targets for metastasis is through advanced computational modeling allowing for the integration of key biological findings with the power of advanced computational measurements and calculations [94]. This method permits the study of the numerous cellular effects and molecular interactions simultaneously and is beginning to increase in popularity [94,103,104,105,106,107]. Araujo et al. [94] developed a model that considered osteoblasts (MC3T3), osteoclasts, precursor osteoblasts, precursor osteoclasts, MSCs, and prostate cancer cells. The authors demonstrated that MSC recruitment is a vital step in the formation of metastatic lesions and that the growth rate calculated using this model was comparable to in vivo experiments, therefore outlining the utility of their computational model. Computational models, such as this one, are becoming more common with advancing technologies. It is our opinion that use of these models may surpass those of in vivo and classic in vitro models in the future; however, this appears to still be in the early stages. It is important to note the major limitation to in silico models being the inability to recapitulate the native physiology. In particular, the effects that bone-specific hormones have on the global physiology are not taken into account [108]. 

## 5. Conclusions

Significant progress has been made in the regeneration of a metastatic bone environment in vivo, but several barriers still exist. The major barriers include the use of intracardiac injections and the use of immunocompromised animals. The adaptation of using tissue-engineered constructs may eventually lead to the ideal model. Future research should focus on using nonreactive tissue-engineered implants to create a humanized environment, without invoking a host immune response. Furthermore, the ability to inject cancer cells of choice in more of an anatomic position in an orthotopic model (e.g., the mammary fat pad for breast cancer) would allow for the creation of a more translatable in vivo model.

## Figures and Tables

**Table 1:** Intraosseous models.

Study	Cell Line Used	Cancer Type	Animal Used	Methodology
Ooi et al. [58]	MCF-7	Breast	Nude mice	Injected into anterior tuberosity of proximal tibia in both limbs
Le Gall et al. [59]	BT474	Breast	Nude mice	Cells injected into tibial marrow canal
Zheng et al. [60]	MCF-7	Breast	Nude mice	Cells injected into tibial marrow canal
Fradet et al. [29]	PC3	Prostate	SCID mice	Cells injected into tibial marrow canal
Akech et al. [61]	PC3	Prostate	SCID mice	Cells injected into tibial marrow canal
Simmons et al. [62]	Probasco	Prostate	Nude mice	Cells injected into tibial marrow canal

**Table 2:** Intracardiac/intravenous models.

Study	Cell Line Used	Cancer Type	Animal Used	Methodology and Outcomes
Le Gall et al. [59]	B02	Breast	Nude mice	B02 cells were injected into the tail vein
Yoneda et al. [63]	MDA-MB-231	Breast	Nude Mice	Spread was mostly to the bone, but occasionally to adrenal glands, ovary, and brain 3–4 weeks after inoculation.
Henriksen et al. [64]	MT-1	Breast	Nude rats	N/A
Yi et al. [65]	MCF-7	Breast	Nude mice	N/A
Canon et al. [66]	MDA-MB-231	Breast	Nude mice	Cells were luciferase labeled
Wu et al. [67]	LNCaP, C4-2, or PC3	Prostate	Athymic or SCID mice	C4-2 cells demonstrated a preference to spinal and lymph node metastases, PC3 cells developed distant widespread metastases, and LNCaP did not demonstrate any metastasess.

**Table 3:** Immunocompetent models.

Study	Cell Line Used	Cancer Type	Animal Used	Methodology and Outcomes
Power et al. [76]	RM1	Prostate	C57Bl/6 mice	Demonstrated no preference for particular bone sites
Ruttinger et al. [77]	P2 and 4T1	Melanoma and Breast	C57Bl/6 and BALB/c mice	Studied tumor regression with anti-CD3 activated and IL-2 expanded tumor vaccine
Arguello et al. [78]	B16	Melanoma	C57Bl/6 mice	Injection sites include left ventricle and mouse tail vein
Lelekakis et al. [81]	4T1	Breast	BALB/c mice	Cells injected into the mammary fat pad
Rabbani et al. [79]	Dunning R3227 Mat Ly Lu	Prostate	Copenhagen rats	Cells injected into left ventricle resulted in tumor metastasis to the lumbar vertebra
Shukeir et al. [69]	Dunning R3327 Mat Ly Lu-PTHrP-8	Prostate	Copenhagen rats	Cells injected into left ventricle resulting in hind limb paralysis from tumor metastasis to the lumbar vertebra

**Table 4:** Humanized models.

Study	Cell Line Used	Cancer Type	Animal Used	Scaffold Source	Injection Site
Shtivelman et al. [83]	NCI-N417, NCI-H82, NCI-H446, NCI-H146, NCI-H345, and NCI-H69	Lung	SCID-hu mice	Human fetal femurs and tibias	Intravenous
Nemeth et al. [84]	DU145, LNCaP, and PC3	Prostate	SCID-hu mice	Human fetal human bone fragments	Intravenous or directly into the target tissue
Yonou et al. [85]	LNCaP and PC3	Prostate	NOD/SCID mice	Human adult cancellous rib fragments from lung cancer patients	Intravenous
Kuperwasser et al. [86]	SUM1315 or PC3	Breast and prostate	NOD/SCID mice	Human bone used from discarded femoral heads from patients undergoing total hip replacement	Intravenous or orthotopic
Yang et al. [87]	GFP-MDA-MB-231	Breast	NOD/SCID mice	Morselized human bone implants	Intravenous
Xia et al. [88]	SUM1315	Breast	NOD/SCID-hu mice	Female human bone tissues were obtained from discarded femoral heads from patients undergoing total hip replacement	Orthotopic

**Table 5:** Tissue-engineered models.

Study	Cell Line Used	Cancer Type	Animal Used	Scaffolds and Methodology	Injection Technique
Moreau et al. [89]	SUM1315	Breast	NOD/SCID mice	Silk fibrin scaffolds coupled with BMP-2 and human bone marrow stromal cells were used	Orthotopic
Schuster et al. [90]	PC3 and H460	Prostate and Lung	SCID mice	Mature osteoblasts were loaded on hydroxyapatite-coated collagen sponges	Percutaneous into bone
Thibaudeau et al. [10]	MDA-MB-231	Breast	NOD/SCID mice	Human osteoblast cell-seeded melt electrospun polycaprolactone scaffolds + recombinant human BMP-7	Intracardiac

## References

[1] Li S., Peng Y., Weinhandl E.D., Blaes A.H., Cetin K., Chia V.M., Stryker S., Pinzone J.J., Acquavella J.F., Arneson T.J. (2012). Estimated number of prevalent cases of metastatic bone disease in the US adult population. Clin. Epidemiol..

[2] Suva L.J., Washam C., Nicholas R.W., Griffin R.J. (2011). Bone metastasis: Mechanisms and therapeutic opportunities. Nat. Rev. Endocrinol..

[3] Society A.C. Cancer Facts & Figures 2017. https://www.cancer.org/research/cancer-facts-statistics/all-cancer-facts-figures/cancer-facts-figures-2017.html.

[4] Quayle L., Ottewell P.D., Holen I. (2015). Bone metastasis: Molecular mechanisms implicated in tumour cell dormancy in breast and prostate cancer. Curr. Cancer Drug Targets.

[5] Holen I., Nutter F., Wilkinson J.M., Evans C.A., Avgoustou P., Ottewell P.D. (2015). Human breast cancer bone metastasis in vitro and in vivo: A novel 3D model system for studies of tumour cell-bone cell interactions. Clin. Exp. Metastasis.

[6] Buijs J.T., van der Pluijm G. (2009). Osteotropic cancers: From primary tumor to bone. Cancer Lett..

[7] Schulman K.L., Kohles J. (2007). Economic burden of metastatic bone disease in the U.S. Cancer.

[8] Morton J.J., Bird G., Refaeli Y., Jimeno A. (2016). Humanized mouse xenograft models: Narrowing the tumor-microenvironment gap. Cancer Res..

[9] Thibaudeau L., Quent V.M., Holzapfel B.M., Taubenberger A.V., Straub M., Hutmacher D.W. (2014). Mimicking breast cancer-induced bone metastasis in vivo: Current transplantation models and advanced humanized strategies. Cancer Metastasis Rev..

[10] Thibaudeau L., Taubenberger A.V., Holzapfel B.M., Quent V.M., Fuehrmann T., Hesami P., Brown T.D., Dalton P.D., Power C.A., Hollier B.G. (2014). A tissue-engineered humanized xenograft model of human breast cancer metastasis to bone. Dis. Models Mech..

[11] Paget S. (1989). The distribution of secondary growths in cancer of the breast. 1889. Cancer Metastasis Rev..

[12] Schilling D., Todenhofer T., Hennenlotter J., Schwentner C., Fehm T., Stenzl A. (2012). Isolated, disseminated and circulating tumour cells in prostate cancer. Nat. Rev. Urol..

[13] Butler T.P., Gullino P.M. (1975). Quantitation of cell shedding into efferent blood of mammary adenocarcinoma. Cancer Res..

[14] Allan A.L., Keeney M. (2010). Circulating tumor cell analysis: Technical and statistical considerations for application to the clinic. J. Oncol..

[15] Campbell J.P., Karolak M.R., Ma Y., Perrien D.S., Masood-Campbell S.K., Penner N.L., Munoz S.A., Zijlstra A., Yang X., Sterling J.A. (2012). Stimulation of host bone marrow stromal cells by sympathetic nerves promotes breast cancer bone metastasis in mice. PLoS Biol..

[16] Cackowski F.C., Taichman R.S. (2018). Parallels between hematopoietic stem cell and prostate cancer disseminated tumor cell regulation. Bone.

[17] Suzman D.L., Boikos S.A., Carducci M.A. (2014). Bone-targeting agents in prostate cancer. Cancer Metastasis Rev..

[18] Wang H., Yu C., Gao X., Welte T., Muscarella A.M., Tian L., Zhao H., Zhao Z., Du S., Tao J. (2015). The osteogenic niche promotes early-stage bone colonization of disseminated breast cancer cells. Cancer Cell.

[19] Zheng H., Kang Y. (2015). Cradle of evil: Osteogenic niche for early bone metastasis. Cancer Cell.

[20] Ghajar C.M., Peinado H., Mori H., Matei I.R., Evason K.J., Brazier H., Almeida D., Koller A., Hajjar K.A., Stainier D.Y. (2013). The perivascular niche regulates breast tumour dormancy. Nat. Cell Biol..

[21] Giancotti F.G. (2013). Mechanisms governing metastatic dormancy and reactivation. Cell.

[22] Linde N., Fluegen G., Aguirre-Ghiso J.A. (2016). The relationship between dormant cancer cells and their microenvironment. Adv. Cancer Res..

[23] Johnson R.W., Finger E.C., Olcina M.M., Vilalta M., Aguilera T., Miao Y., Merkel A.R., Johnson J.R., Sterling J.A., Wu J.Y. (2016). Erratum: Induction of LIFR confers a dormancy phenotype in breast cancer cells disseminated to the bone marrow. Nat. Cell Biol..

[24] Kobayashi A., Okuda H., Xing F., Pandey P.R., Watabe M., Hirota S., Pai S.K., Liu W., Fukuda K., Chambers C. (2011). Bone morphogenetic protein 7 in dormancy and metastasis of prostate cancer stem-like cells in bone. J. Exp. Med..

[25] Gawrzak S., Rinaldi L., Gregorio S., Arenas E.J., Salvador F., Urosevic J., Figueras-Puig C., Rojo F., Del Barco Barrantes I., Cejalvo J.M. (2018). MSK1 regulates luminal cell differentiation and metastatic dormancy in ER(+) breast cancer. Nat. Cell Biol..

[26] Yu-Lee L.Y., Yu G., Lee Y.C., Lin S.C., Pan J., Pan T., Yu K.J., Liu B., Creighton C.J., Rodriguez-Canales J. (2018). Osteoblast-secreted factors mediate dormancy of metastatic prostate cancer in the bone via activation of the TGFβRIII-p38MAPK-pS249/T252RB pathway. Cancer Res..

[27] Lu Y., Cai Z., Xiao G., Keller E.T., Mizokami A., Yao Z., Roodman G.D., Zhang J. (2007). Monocyte chemotactic protein-1 mediates prostate cancer-induced bone resorption. Cancer Res..

[28] Yasuda H., Shima N., Nakagawa N., Yamaguchi K., Kinosaki M., Mochizuki S., Tomoyasu A., Yano K., Goto M., Murakami A. (1998). Osteoclast differentiation factor is a ligand for osteoprotegerin/osteoclastogenesis-inhibitory factor and is identical to trance/RANKL. Proc. Natl. Acad. Sci. USA.

[29] Fradet A., Sorel H., Depalle B., Serre C.M., Farlay D., Turtoi A., Bellahcene A., Follet H., Castronovo V., Clezardin P. (2013). A new murine model of osteoblastic/osteolytic lesions from human androgen-resistant prostate cancer. PLoS ONE.

[30] Lipton A., Small E., Saad F., Gleason D., Gordon D., Smith M., Rosen L., Kowalski M.O., Reitsma D., Seaman J. (2002). The new bisphosphonate, zometa (zoledronic acid), decreases skeletal complications in both osteolytic and osteoblastic lesions: A comparison to pamidronate. Cancer Investig..

[31] Fizazi K., Carducci M., Smith M., Damiao R., Brown J., Karsh L., Milecki P., Shore N., Rader M., Wang H. (2011). Denosumab versus zoledronic acid for treatment of bone metastases in men with castration-resistant prostate cancer: A randomised, double-blind study. Lancet.

[32] Holen I., Coleman R.E. (2010). Bisphosphonates as treatment of bone metastases. Curr. Pharm. Des..

[33] Luftner D., Niepel D., Steger G.G. (2018). Therapeutic approaches for protecting bone health in patients with breast cancer. Breast.

[34] Kingsley L.A., Fournier P.G., Chirgwin J.M., Guise T.A. (2007). Molecular biology of bone metastasis. Mol. Cancer Ther..

[35] Kakonen S.M., Selander K.S., Chirgwin J.M., Yin J.J., Burns S., Rankin W.A., Grubbs B.G., Dallas M., Cui Y., Guise T.A. (2002). Transforming growth factor-beta stimulates parathyroid hormone-related protein and osteolytic metastases via smad and mitogen-activated protein kinase signaling pathways. J. Biol. Chem..

[36] Yin J.J., Selander K., Chirgwin J.M., Dallas M., Grubbs B.G., Wieser R., Massague J., Mundy G.R., Guise T.A. (1999). TGF-beta signaling blockade inhibits PTHrP secretion by breast cancer cells and bone metastases development. J. Clin. Investig..

[37] Kitazawa S., Kitazawa R. (2002). Rank ligand is a prerequisite for cancer-associated osteolytic lesions. J. Pathol..

[38] McCabe N.P., Kerr B.A., Madajka M., Vasanji A., Byzova T.V. (2011). Augmented osteolysis in SPARC-deficient mice with bone-residing prostate cancer. Neoplasia.

[39] Bendre M.S., Margulies A.G., Walser B., Akel N.S., Bhattacharrya S., Skinner R.A., Swain F., Ramani V., Mohammad K.S., Wessner L.L. (2005). Tumor-derived interleukin-8 stimulates osteolysis independent of the receptor activator of nuclear factor-κB ligand pathway. Cancer Res..

[40] Lu Y., Chen Q., Corey E., Xie W., Fan J., Mizokami A., Zhang J. (2009). Activation of MCP-1/CCR2 axis promotes prostate cancer growth in bone. Clin. Exp. Metastasis.

[41] Gunalp B., Oner A.O., Ince S., Alagoz E., Ayan A., Arslan N. (2015). Evaluation of radiographic and metabolic changes in bone metastases in response to systemic therapy with ^18^FDG-PET/CT. Radiol. Oncol..

[42] Dai J., Hensel J., Wang N., Kruithof-de Julio M., Shiozawa Y. (2016). Mouse models for studying prostate cancer bone metastasis. BoneKEy Rep..

[43] Rutkovskiy A., Stenslokken K.O., Vaage I.J. (2016). Osteoblast differentiation at a glance. Med. Sci. Monit. Basic Res..

[44] Fradet A., Bouchet M., Delliaux C., Gervais M., Kan C., Benetollo C., Pantano F., Vargas G., Bouazza L., Croset M. (2016). Estrogen related receptor alpha in castration-resistant prostate cancer cells promotes tumor progression in bone. Oncotarget.

[45] Dai J., Hall C.L., Escara-Wilke J., Mizokami A., Keller J.M., Keller E.T. (2008). Prostate cancer induces bone metastasis through WNT-induced bone morphogenetic protein-dependent and independent mechanisms. Cancer Res..

[46] Hall J.M., Korach K.S. (2003). Stromal cell-derived factor 1, a novel target of estrogen receptor action, mediates the mitogenic effects of estradiol in ovarian and breast cancer cells. Mol. Endocrinol..

[47] Neveu B., Jain P., Tetu B., Wu L., Fradet Y., Pouliot F. (2016). A PCa3 gene-based transcriptional amplification system targeting primary prostate cancer. Oncotarget.

[48] Thiele S., Rachner T.D., Rauner M., Hofbauer L.C. (2016). Wnt5A and its receptors in the bone-cancer dialogue. J. Bone Miner. Res..

[49] Chen G., Shukeir N., Potti A., Sircar K., Aprikian A., Goltzman D., Rabbani S.A. (2004). Up-regulation of WNT-1 and β-catenin production in patients with advanced metastatic prostate carcinoma: Potential pathogenetic and prognostic implications. Cancer.

[50] Huang W.C., Xie Z., Konaka H., Sodek J., Zhau H.E., Chung L.W. (2005). Human osteocalcin and bone sialoprotein mediating osteomimicry of prostate cancer cells: Role of camp-dependent protein kinase a signaling pathway. Cancer Res..

[51] Kerr B.A., McCabe N.P., Feng W., Byzova T.V. (2013). Platelets govern pre-metastatic tumor communication to bone. Oncogene.

[52] Singh A.S., Figg W.D. (2005). In vivo models of prostate cancer metastasis to bone. J. Urol..

[53] Mak I.W., Evaniew N., Ghert M. (2014). Lost in translation: Animal models and clinical trials in cancer treatment. Am. J. Transl. Res..

[54] Price J.E. (2014). Spontaneous and experimental metastasis models: Nude mice. Methods Mol. Biol..

[55] Rosol T.J., Tannehill-Gregg S.H., LeRoy B.E., Mandl S., Contag C.H. (2003). Animal models of bone metastasis. Cancer.

[56] Simmons J.K., Hildreth B.E., Supsavhad W., Elshafae S.M., Hassan B.B., Dirksen W.P., Toribio R.E., Rosol T.J. (2015). Animal models of bone metastasis. Vet. Pathol..

[57] Langlais L.M., Gibson J., Taylor J.A., Caswell J.L. (2006). Pulmonary adenocarcinoma with metastasis to skeletal muscle in a cat. Can. Vet. J..

[58] Ooi L.L., Zheng Y., Zhou H., Trivedi T., Conigrave A.D., Seibel M.J., Dunstan C.R. (2010). Vitamin d deficiency promotes growth of MCF-7 human breast cancer in a rodent model of osteosclerotic bone metastasis. Bone.

[59] Le Gall C., Bellahcene A., Bonnelye E., Gasser J.A., Castronovo V., Green J., Zimmermann J., Clezardin P. (2007). A cathepsin k inhibitor reduces breast cancer induced osteolysis and skeletal tumor burden. Cancer Res..

[60] Zheng Y., Zhou H., Fong-Yee C., Modzelewski J.R., Seibel M.J., Dunstan C.R. (2008). Bone resorption increases tumour growth in a mouse model of osteosclerotic breast cancer metastasis. Clin. Exp. Metastasis.

[61] Akech J., Wixted J.J., Bedard K., van der Deen M., Hussain S., Guise T.A., van Wijnen A.J., Stein J.L., Languino L.R., Altieri D.C. (2010). Runx2 association with progression of prostate cancer in patients: Mechanisms mediating bone osteolysis and osteoblastic metastatic lesions. Oncogene.

[62] Simmons J.K., Dirksen W.P., Hildreth B.E., Dorr C., Williams C., Thomas R., Breen M., Toribio R.E., Rosol T.J. (2014). Canine prostate cancer cell line (probasco) produces osteoblastic metastases in vivo. Prostate.

[63] Yoneda T., Williams P.J., Hiraga T., Niewolna M., Nishimura R. (2001). A bone-seeking clone exhibits different biological properties from the MDA-MB-231 parental human breast cancer cells and a brain-seeking clone in vivo and in vitro. J. Bone Miner. Res..

[64] Henriksen G., Breistol K., Bruland O.S., Fodstad O., Larsen R.H. (2002). Significant antitumor effect from bone-seeking, alpha-particle-emitting ^223^Ra demonstrated in an experimental skeletal metastases model. Cancer Res..

[65] Yi B., Williams P.J., Niewolna M., Wang Y., Yoneda T. (2002). Tumor-derived platelet-derived growth factor-bb plays a critical role in osteosclerotic bone metastasis in an animal model of human breast cancer. Cancer Res..

[66] Canon J.R., Roudier M., Bryant R., Morony S., Stolina M., Kostenuik P.J., Dougall W.C. (2008). Inhibition of rankl blocks skeletal tumor progression and improves survival in a mouse model of breast cancer bone metastasis. Clin. Exp. Metastasis.

[67] Wu T.T., Sikes R.A., Cui Q., Thalmann G.N., Kao C., Murphy C.F., Yang H., Zhau H.E., Balian G., Chung L.W. (1998). Establishing human prostate cancer cell xenografts in bone: Induction of osteoblastic reaction by prostate-specific antigen-producing tumors in athymic and SCID/bg mice using LNCaP and lineage-derived metastatic sublines. Int. J. Cancer.

[68] Wu X., Gong S., Roy-Burman P., Lee P., Culig Z. (2013). Current mouse and cell models in prostate cancer research. Endocr. Relat. Cancer.

[69] Shukeir N., Arakelian A., Chen G., Garde S., Ruiz M., Panchal C., Rabbani S.A. (2004). A synthetic 15-mer peptide (PCK3145) derived from prostate secretory protein can reduce tumor growth, experimental skeletal metastases, and malignancy-associated hypercalcemia. Cancer Res..

[70] Walsh M.C., Kim N., Kadono Y., Rho J., Lee S.Y., Lorenzo J., Choi Y. (2006). Osteoimmunology: Interplay between the immune system and bone metabolism. Annu. Rev. Immunol..

[71] Rho J., Takami M., Choi Y. (2004). Osteoimmunology: Interactions of the immune and skeletal systems. Mol. Cells.

[72] Okamoto K., Takayanagi H. (2018). Osteoimmunology. Cold Spring Harb. Perspect. Med..

[73] Capietto A.H., Faccio R. (2014). Immune regulation of bone metastasis. BoneKEy Rep..

[74] Sallusto F., Lanzavecchia A. (2000). Understanding dendritic cell and T-lymphocyte traffic through the analysis of chemokine receptor expression. Immunol. Rev..

[75] Huang A.Y., Golumbek P., Ahmadzadeh M., Jaffee E., Pardoll D., Levitsky H. (1994). Role of bone marrow-derived cells in presenting MHC class I-restricted tumor antigens. Science.

[76] Power C.A., Pwint H., Chan J., Cho J., Yu Y., Walsh W., Russell P.J. (2009). A novel model of bone-metastatic prostate cancer in immunocompetent mice. Prostate.

[77] Ruttinger D., Li R., Urba W.J., Fox B.A., Hu H.M. (2003). Evaluation of a preclinical model of bone metastasis for the study of adoptive immunotherapy. Eur. Surg. Res..

[78] Arguello F., Baggs R.B., Frantz C.N. (1988). A murine model of experimental metastasis to bone and bone marrow. Cancer Res..

[79] Rabbani S.A., Harakidas P., Bowlin T., Attardo G. (1998). Effect of nucleoside analogue bch-4556 on prostate cancer growth and metastases in vitro and in vivo. Cancer Res..

[80] Wright L.E., Ottewell P.D., Rucci N., Peyruchaud O., Pagnotti G.M., Chiechi A., Buijs J.T., Sterling J.A. (2016). Murine models of breast cancer bone metastasis. BoneKEy Rep..

[81] Lelekakis M., Moseley J.M., Martin T.J., Hards D., Williams E., Ho P., Lowen D., Javni J., Miller F.R., Slavin J. (1999). A novel orthotopic model of breast cancer metastasis to bone. Clin. Exp. Metastasis.

[82] Aslakson C.J., Miller F.R. (1992). Selective events in the metastatic process defined by analysis of the sequential dissemination of subpopulations of a mouse mammary tumor. Cancer Res..

[83] Shtivelman E., Namikawa R. (1995). Species-specific metastasis of human tumor cells in the severe combined immunodeficiency mouse engrafted with human tissue. Proc. Natl. Acad. Sci. USA.

[84] Nemeth J.A., Harb J.F., Barroso U., He Z., Grignon D.J., Cher M.L. (1999). Severe combined immunodeficient-hu model of human prostate cancer metastasis to human bone. Cancer Res..

[85] Yonou H., Yokose T., Kamijo T., Kanomata N., Hasebe T., Nagai K., Hatano T., Ogawa Y., Ochiai A. (2001). Establishment of a novel species- and tissue-specific metastasis model of human prostate cancer in humanized non-obese diabetic/severe combined immunodeficient mice engrafted with human adult lung and bone. Cancer Res..

[86] Kuperwasser C., Dessain S., Bierbaum B.E., Garnet D., Sperandio K., Gauvin G.P., Naber S.P., Weinberg R.A., Rosenblatt M. (2005). A mouse model of human breast cancer metastasis to human bone. Cancer Res..

[87] Yang W., Lam P., Kitching R., Kahn H.J., Yee A., Aubin J.E., Seth A. (2007). Breast cancer metastasis in a human bone nod/scid mouse model. Cancer Biol. Ther..

[88] Xia T.S., Wang G.Z., Ding Q., Liu X.A., Zhou W.B., Zhang Y.F., Zha X.M., Du Q., Ni X.J., Wang J. (2012). Bone metastasis in a novel breast cancer mouse model containing human breast and human bone. Breast Cancer Res. Treat..

[89] Moreau J.E., Anderson K., Mauney J.R., Nguyen T., Kaplan D.L., Rosenblatt M. (2007). Tissue-engineered bone serves as a target for metastasis of human breast cancer in a mouse model. Cancer Res..

[90] Schuster J., Zhang J., Longo M. (2006). A novel human osteoblast-derived severe combined immunodeficiency mouse model of bone metastasis. J. Neurosurg. Spine.

[91] Marlow R., Honeth G., Lombardi S., Cariati M., Hessey S., Pipili A., Mariotti V., Buchupalli B., Foster K., Bonnet D. (2013). A novel model of dormancy for bone metastatic breast cancer cells. Cancer Res..

[92] Bragado P., Sosa M.S., Keely P., Condeelis J., Aguirre-Ghiso J.A. (2012). Microenvironments dictating tumor cell dormancy. Recent Results Cancer Res..

[93] Walker N.D., Patel J., Munoz J.L., Hu M., Guiro K., Sinha G., Rameshwar P. (2016). The bone marrow niche in support of breast cancer dormancy. Cancer Lett..

[94] Araujo A., Cook L.M., Lynch C.C., Basanta D. (2014). An integrated computational model of the bone microenvironment in bone-metastatic prostate cancer. Cancer Res..

[95] Skardal A., Devarasetty M., Forsythe S., Atala A., Soker S. (2016). A reductionist metastasis-on-a-chip platform for in vitro tumor progression modeling and drug screening. Biotechnol. Bioeng..

[96] Carvalho M.R., Reis R.L., Oliveira J.M. (2018). Mimicking the 3D biology of osteochondral tissue with microfluidic-based solutions: Breakthroughs towards boosting drug testing and discovery. Drug Discov. Today.

[97] Hao S., Ha L., Cheng G., Wan Y., Xia Y., Sosnoski D.M., Mastro A.M., Zheng S.Y. (2018). A spontaneous 3D bone-on-a-chip for bone metastasis study of breast cancer cells. Small.

[98] Narkhede A.A., Shevde L.A., Rao S.S. (2017). Biomimetic strategies to recapitulate organ specific microenvironments for studying breast cancer metastasis. Int. J. Cancer.

[99] Kong J., Luo Y., Jin D., An F., Zhang W., Liu L., Li J., Fang S., Li X., Yang X. (2016). A novel microfluidic model can mimic organ-specific metastasis of circulating tumor cells. Oncotarget.

[100] Garzon-Alvarado D.A. (2012). A mathematical model for describing the metastasis of cancer in bone tissue. Comput. Methods Biomech. Biomed. Eng..

[101] Bersini S., Jeon J.S., Dubini G., Arrigoni C., Chung S., Charest J.L., Moretti M., Kamm R.D. (2014). A microfluidic 3D in vitro model for specificity of breast cancer metastasis to bone. Biomaterials.

[102] Jeon J.S., Bersini S., Gilardi M., Dubini G., Charest J.L., Moretti M., Kamm R.D. (2015). Human 3D vascularized organotypic microfluidic assays to study breast cancer cell extravasation. Proc. Natl. Acad. Sci. USA.

[103] Munoz A.I., Tello J.I. (2017). On a mathematical model of bone marrow metastatic niche. Math. Biosci. Eng..

[104] Zhou X., Liu J. (2014). A computational model to predict bone metastasis in breast cancer by integrating the dysregulated pathways. BMC Cancer.

[105] Newton P.K., Mason J., Venkatappa N., Jochelson M.S., Hurt B., Nieva J., Comen E., Norton L., Kuhn P. (2015). Spatiotemporal progression of metastatic breast cancer: A markov chain model highlighting the role of early metastatic sites. NPJ Breast Cancer.

[106] Gallaher J., Cook L.M., Gupta S., Araujo A., Dhillon J., Park J.Y., Scott J.G., Pow-Sang J., Basanta D., Lynch C.C. (2014). Improving treatment strategies for patients with metastatic castrate resistant prostate cancer through personalized computational modeling. Clin. Exp. Metastasis.

[107] Hayashi N., Iwamoto T., Qi Y., Niikura N., Santarpia L., Yamauchi H., Nakamura S., Hortobagyi G.N., Pusztai L., Symmans W.F. (2017). Bone metastasis-related signaling pathways in breast cancers stratified by estrogen receptor status. J. Cancer.

[108] Mera P., Ferron M., Mosialou I. (2017). Regulation of energy metabolism by bone-derived hormones. Cold Spring Harb. Perspect. Med..

